# Case series: Diffusion weighted MRI appearance in prostatic abscess

**DOI:** 10.4103/0971-3026.76054

**Published:** 2011

**Authors:** Paramjeet Singh, Mukesh K Yadav, Shrawan K Singh, Anupam Lal, Niranjan Khandelwal

**Affiliations:** Department of Radiodiagnosis, Postgraduate Institute of Medical Education and Research, Chandigarh, India; 1 Department of Urology, Postgraduate Institute of Medical Education and Research, Chandigarh, India

**Keywords:** Apparent diffusion coefficient, diffusion-weighted imaging, magnetic resonance imaging, prostatic abscess, transrectal ultrasound

## Abstract

Diffusion: weighted MRI (DWI) is a novel technique that analyzes the diffusion of water molecules *in vivo*. DWI has been used extensively in the central nervous system. Its use in body imaging is on the rise. In the prostate, it has been used in the evaluation of prostatic carcinoma. We present DWI findings in two patients of prostatic abscess.

## Introduction

Prostatic abscesses are rare entities. Transrectal ultrasonography (TRUS) is the initial investigation of choice.[1] Conventional MRI has also been used for this.[2] Diffusion-weighted MRI (DWI) is a relatively new MRI technique that evaluates molecular diffusion at the cellular level.[3] It is a useful technique in the central nervous system.[4–6] Its use has also been described in the prostate,[7] uterus[8] and ovary.[9] In the prostate, it has been primarily used for the evaluation of prostatic carcinoma.[7] DWI has not been described previously in prostatic abscess. We present the DWI findings in two patients with prostatic infection.

## Case Report

**Figure 1 (A-E):**
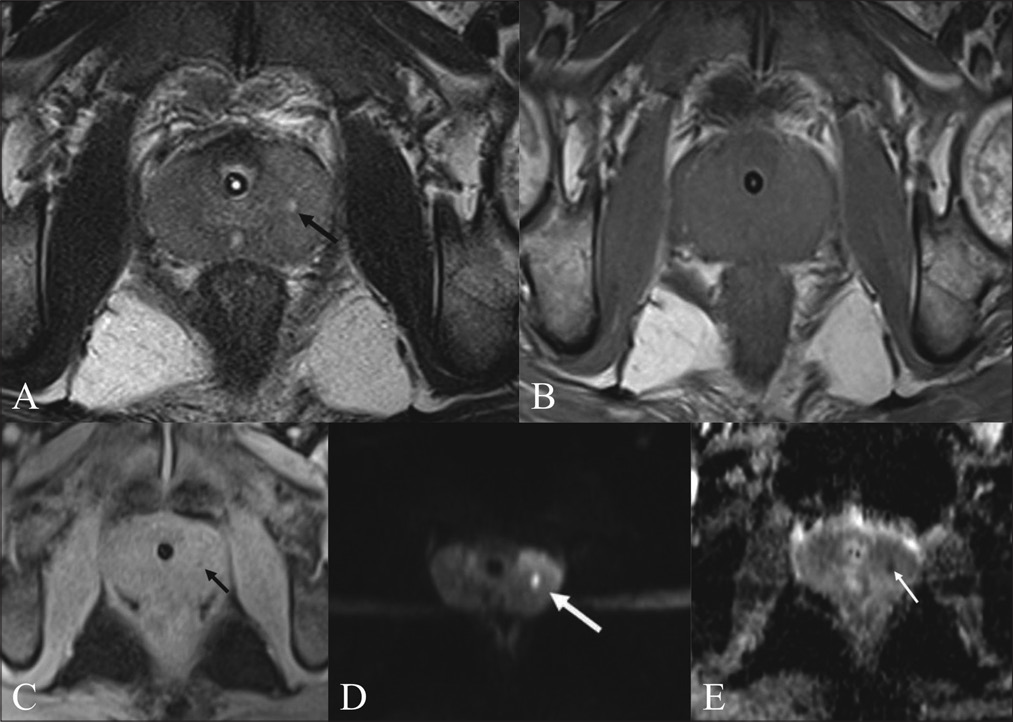
Prostatic abscess – case 1. Axial T2W image (A) of the prostate shows a focus of hyperintense signal (arrow) in the left midzone of the peripheral gland. On the axial T1W image (B), the lesion is barely seen. An axial, contrast-enhanced, T1W image (C) shows that the lesion (arrow) has peripheral enhancement and central non enhancement. DWI (b value=800) (D) shows high signal in the lesion (arrow) due to restriction of diffusion. The corresponding ADC map (E) shows low signal (arrow)

A 22-year-old male presented with acute retention of urine and an enlarged and tender prostate. There was no history of sexual contact and human immunodeficiency virus (HIV) serology was negative. Urine examination showed 20 pus cells/hpf. TRUS revealed ill-defined hypoechoic areas in the peripheral zone of the prostate. MRI was performed using T2W fast spin-echo, pre- and postgadolinium-enhanced T1W spin-echo and DWI sequences on a 3.0- Tesla MRI unit (Verio; Siemens, Erlangen, Germany). For DWI, we used a single-shot echo- planar imaging EPI sequence with TR/TE/3000/79 ms, b factors of 50/400/800 s/mm^2^, 10 averages, 4-mm slices with a parallel acquisition technique PAT factor of 2 (generalized autocalibrating partial parallel acquisition–GRAPPA) leading to a time of 4.41 min for acquisition of a three-directional trace and apparent diffusion coefficient (ADC) maps. It revealed multiple foci of a T2-hyperintense signal [Figure 1A] in the peripheral part of the prostate, poorly visualized on T1W images [Figure 1B] with peripheral enhancement on postgadolinium images [Figure 1C]. The DWI revealed diffusion restriction in the lesions [Figure 1D]. The corresponding ADC map revealed low signal [Figure 1E], with mean values of 0.63 ± 0.07 × 10^-3^ mm^2^/s, using an average of 5 ROIs of 0.08 sq cm each. The urine culture was sterile. The patient improved after administration of antibiotics (ofloxacin) for 4 weeks.

**Figure 2 (A-E):**
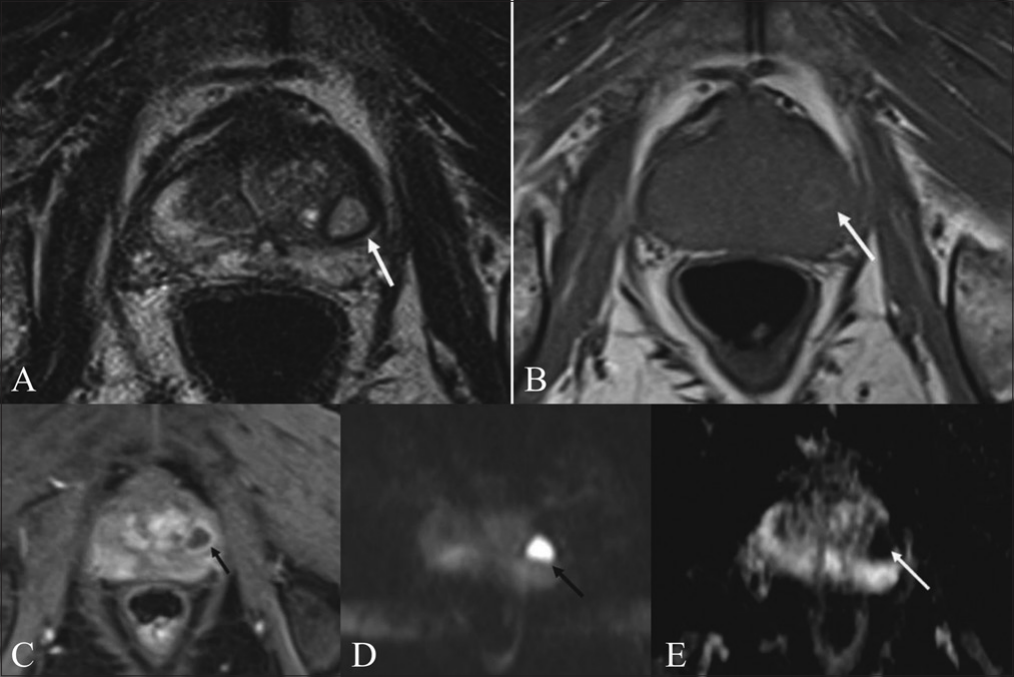
Prostatic abscess – case 2. Axial T2W MRI of the prostate (A) shows high signal (arrow) in the central gland, in the left midzone. Axial T1W image (B) shows mixed signal intensity with peripheral hyperintensity (arrow). Axial, contrast-enhanced T1W image (C) shows a peripherally enhancing abscess (arrow). DWI (b=800) (D) shows restriction of diffusion (arrow) in the lesion. The corresponding ADC map (E) shows low signal (arrow)

**Figure 3 (A,B):**
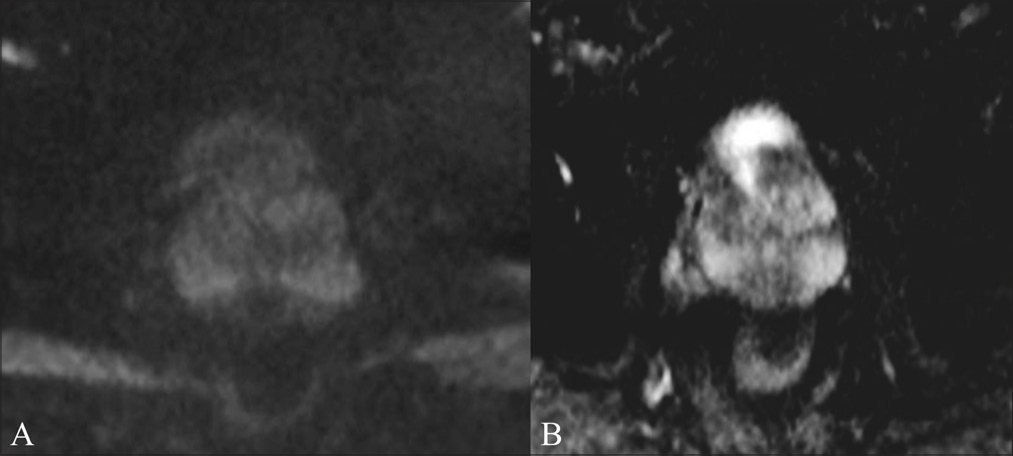
Follow up images of case 2 after 4 months. DWI (b=800) (A) shows resolution of the previously shown lesion. Normalization of signal is also seen in the respective ADC map (B)

Our second patient, a 62-year-old diabetic man, presented with dysuria and high-grade fever for 10 days. He had an enlarged and tender prostate, an elevated total leucocyte count and 10–12 pus cells/hpf in the urine. TRUS revealed a small heterogeneous area of altered echogenecity in the central gland in the left midzone. On TRUS, infection and neoplasm were both considered in the differential diagnosis. His prostate-specific antigen (PSA) level was 6.3 ng/ml. MRI, using the same sequences as in the first patient, revealed a T2-hyperintense signal [Figure 2A] in the central gland in the left midzone, barely appreciated on T1W images [Figure 2B], with peripheral enhancement suggesting an abscess [Figure 2C]. On DWI, the lesion appeared bright due to the restriction of diffusion [Figure 2D]. A low signal was seen on the ADC map [Figure 2E] (mean ADC values. -0.61 ± 0.06 × 10^-3^). The urine culture grew *E. coli*. The patient improved clinically after treatment with antibiotic (prolifloxacin). The patient was clinically normal over a 4-month follow-up, with a reducing serial PSA level. A repeat MRI carried out after 4 months revealed no diffusion restriction [Figure 3A] in the previously abnormal area, with no definite low signal in the corresponding ADC map [Figure 3B].

## Discussion

Prostatic abscess is an uncommon entity usually caused by *E. coli* and Staphylococci.[10,11] Common presenting features are dysuria, fever, suprapubic pain and/or urinary retention. Urine examination usually reveals pus cells. TRUS is the imaging modality of choice and reveals ill-defined hypoechoic areas within an enlarged or distorted gland. TRUS has a good sensitivity for diagnosing large, walled-off abscesses but, in the initial stages of abscess formation, it may be inconclusive. MRI shows a hypointense signal on T1W and a hyperintense signal on T2W images,[2] with peripheral contrast enhancement.

DWI has not been evaluated for prostatic abscess till now. It is a novel technique that analyzes the diffusion of water molecules *in vivo*[3] and has been used in the central nervous system[4–6] as well as for uterine,[8] ovarian[9] and prostatic[7] pathologies.

DWI has been used in the prostate mainly in prostatic carcinoma.[7] The significant difference in ADC values between the cancerous part of the prostate and the normal peripheral zone has been described by various authors.[7] The mean ADC values are lower in prostatic carcinoma as compared with the normal peripheral zone.[7]

We have described two patients of prostatic abscess confirmed by urine examination/culture. The lesions in both of them showed restriction of diffusion corresponding to hypoechoic lesions on TRUS. In one patient, there were multiple small foci of abscesses while the second patient had a large single cavity. The area of diffusion restriction corresponded to the area of T2 abnormality as well as the enhancement thus more specifically representing pus formation.

The mean ADC values in the abscesses were very low (0.61–0.63 × 10^-3^ mm^2^/s) compared not only with normal published values of the peripheral zone (1.57–1.82 × 10^-3^mm ^2^/s) but also with cancerous tissue (0.93–1.43 × 10^-3^mm^2^/s). In our experience, the corresponding values are 1.41 ± 0.23 × 10^-3^ mm^2^/s and 0.9 ± 0.17 × 10^-3^mm^2^/s, respectively [unpublished data]).

Our aim was to highlight the DWI findings in prostatic abscesses. Whether this has any relevance in the management of such patients or can help differentiate abscesses from carcinoma in elderly patients is difficult to comment upon and needs further analysis.

## References

[1] Oliveira P, Andrade JA, Porto HC, Filho JE, Vinhaes AF (2003). Diagnosis and treatment of prostatic abscess. Int Braz J Urol.

[2] Papanicolaou N, Pfister RC, Stafford SA, Parkhurst EC (1987). Prostatic abscess: imaging with transrectalsonography and MR. AJR Am J Roentgenol.

[3] Le Bihan D, Breton E, Lallemand D, Grenier P, Cabanis E, Laval-Jeantet M (1986). MR imaging of intravoxel incoherent motions: application to diffusion and perfusion in neurologic disorders. Radiology.

[4] Ebisu T, Tanaka C, Umeda M, Kitamura M, Naruse S, Higuchi T (1996). Discrimination of brain abscess from necrotic or cystic tumors by diffusion-weighted echo planar imaging. Magn Reson Imaging.

[5] Warach S, Gaa J, Siewert B, Wielopolski P, Edelman RR (1995). Acute human stroke studied by whole brain echo planar diffusion-weighted magnetic resonance imaging. Ann Neurol.

[6] Hakyemez B, Aksoy U, Yildiz H, Ergin N (2005). Intracranial epidermoid cysts.Diffusion-weighted, FLAIR and conventional MR findings. Eur J Radiol.

[7] Hosseinzadeh K, Schwarz SD (2004). Endorectal diffusion-weighted imaging in prostate cancer to differentiate malignant and benign peripheral zone tissue. J MagnReson Imaging.

[8] Tamai K, Koyama T, Saga T, Morisawa N, Fujimoto K, Mikami Y (2008). The utility of diffusion-weighted MR imaging for differentiating uterine sarcomas from benign leiomyomas. Eur Radiol.

[9] Nakayama T, Yoshimitsu K, Irie H, Aibe H, Tajima T, Nishie A (2005). Diffusion-weighted echo-planar MR imaging and ADC mapping in the differential diagnosis of ovarian cystic masses: usefulness of detecting keratinoid substances in mature cystic teratomas. J Magn Reson Imaging.

[10] Dajani AM, O’Flynn JD (1968). Prostatic abscess.A report of 25 cases. Br J Urol.

[11] Meares EM (1988). Urinary tract infections in the male patient. Urology.

